# Measuring symptom burden in patients with cancer during a pandemic: the MD Anderson symptom inventory for COVID-19 (MDASI-COVID)

**DOI:** 10.1186/s41687-023-00591-x

**Published:** 2023-05-26

**Authors:** Loretta A. Williams, Meagan S. Whisenant, Tito R. Mendoza, Angela E. Peek, Donna Malveaux, Donna K. Griffin, Darcy A. Ponce, Bruno Palma Granwehr, Ajay Sheshadri, Katherine A. Hutcheson, Sara M. Ali, Susan K. Peterson, John V. Heymach, Charles S. Cleeland, Ishwaria M. Subbiah

**Affiliations:** 1 Department of Symptom Research, The University of Texas MD Anderson Cancer Center, 1515 Holcombe Boulevard, Unit 1450, Houston, Texas 77030 USA; 2 Department of Behavioral Science, The University of Texas MD Anderson Cancer Center, 1515 Holcombe Blvd, Unit 1330, Houston, TX 77030 USA; 3 Office of Patient-Centered Research Outcomes, Center for Cancer Research, National Cancer Institute, Bldg. 82, Rm. B03A, Bethesda, MD 20892 USA; 4 Department of Electronic Health Record Ambulatory Access & Revenue, The University of Texas MD Anderson Cancer Center, 1515 Holcombe Boulevard, Unit 1746, Houston, TX 77030 USA; 5 Department of Infectious Diseases, The University of Texas MD Anderson Cancer Center, 1515 Holcombe Boulevard, Unit 0402, Houston, TX 77030 USA; 6 Department of Pulmonary Medicine, The University of Texas MD Anderson Cancer Center, 1515 Holcombe Boulevard, Unit 1462, Houston, TX 77030 USA; 7 Department of Head & Neck Surgery, The University of Texas MD Anderson Cancer Center, 1515 Holcombe Boulevard, Unit 1445, Houston, TX 77030 USA; 8 Department of Electronic Health Record Analytics & Reporting, The University of Texas MD Anderson Cancer Center, 1515 Holcombe Boulevard, Unit 1747, Houston, TX 77030 USA; 9 Department of Behavioral Science, The University of Texas MD Anderson Cancer Center, 1515 Holcombe Boulevard, Unit 1330, Houston, TX 77030 USA; 10 Department of Thoracic–Head & Neck Medical Oncology, The University of Texas MD Anderson Cancer Center, 1515 Holcombe Boulevard, Unit 0432, Houston, TX 77030 USA; 11 Symptom Assessment Systems LLC, 1416 Marconi St., Houston, TX 77019 USA; 12 Sarah Cannon Research Institute, 1100 Dr. Martin L. King Jr. Blvd., Suite 800, Nashville, TN 37203 USA

**Keywords:** COVID-19, Electronic patient-reported outcomes, Symptoms, Symptom burden, Cancer, Quality of life

## Abstract

**Background:**

Symptom expression in SARS-CoV-2 infection (COVID-19) may affect patients already symptomatic with cancer. Patient-reported outcomes (PROs) can describe symptom burden during the acute and postacute stages of COVID-19 and support risk stratification for levels of care. At the start of the COVID-19 pandemic, our purpose was to rapidly develop, launch through an electronic patient portal, and provide initial validation for a PRO measure of COVID-19 symptom burden in patients with cancer.

**Methods:**

We conducted a CDC/WHO web-based scan for COVID-19 symptoms and a relevance review of symptoms by an expert panel of clinicians treating cancer patients with COVID-19 to create a provisional MD Anderson Symptom Inventory for COVID-19 (MDASI-COVID). English-speaking adults with cancer who tested positive for COVID-19 participated in the psychometric testing phase. Patients completed longitudinal assessments of the MDASI-COVID and the EuroQOL 5 Dimensions 5 Levels (EQ-5D-5L) utility index and visual analog scale, which were presented through an electronic health record patient portal. To test the validity of the MDASI-COVID to distinguish between known groups of patients, we hypothesized that patients hospitalized, including having a hospitalization extended, for COVID-19 versus those not hospitalized would experience higher symptom burden. Correlation of mean symptom severity and interference scores with relevant EQ-5D-5L scores tested concurrent validity. The reliability of the MDASI-COVID was evaluated by calculating Cronbach alpha coefficients and test-retest reliability was evaluated by calculating Pearson correlation coefficients between the initial assessment and a second assessment no more than 14 days later.

**Results:**

The web-based scan found 31 COVID-19-related symptoms; rankings of a 14-clinician expert panel reduced this list to 11 COVID-specific items to be added to the core MDASI. Time from literature scan start in March 2020 to instrument launch in May 2020 was 2 months. Psychometric analysis established the MDASI-COVID’s reliability, known-group validity, and concurrent validity.

**Conclusions:**

We were able to rapidly develop and electronically launch a PRO measure of COVID-19 symptom burden in patients with cancer. Additional research is needed to confirm the content domain and predictive validity of the MDASI-COVID and define the symptom burden trajectory of COVID-19.

**Supplementary Information:**

The online version contains supplementary material available at 10.1186/s41687-023-00591-x.

## Background

Understanding of the symptom burden associated with the viral infection caused by severe acute respiratory syndrome-associated coronavirus 2 (SARS-CoV-2, also known as COVID-19) continues to evolve. Symptom expression in COVID-19 ranges from asymptomatic to extremely severe and from acute symptoms to symptoms related to persistent postacute sequelae of COVID-19 (PASC; also called long COVID). Prolonged symptom burden and morbidity related to COVID-19 infection are increasingly apparent, especially among adults hospitalized with severe COVID-19 [1].

Common acute symptoms of COVID-19 include fever, cough, myalgia, fatigue, shortness of breath, joint pain, headache, gastrointestinal symptoms, and altered sense of smell and taste [2, 3]. Approximately one-third of adults treated for COVID-19 in the outpatient setting reported that they had not returned to usual health within 2–3 weeks of testing positive, [1] while 87% of patients recovered from COVID-19 had one or more persistent symptoms, commonly fatigue and shortness of breath [3]. Symptoms found more frequently in PASC than in other severe illnesses include loss of smell and taste, memory loss, chest pain, difficulty concentrating, confusion, and bone and joint pain [4]. There is no current consensus on the specific symptoms involved in PASC due to its variable, multisystem nature [5].

Understanding the symptom burden and related health impact of COVID-19 is important for patients with cancer, given that this population is vulnerable to the direct impacts of COVID-19 infection and to delays in cancer diagnosis and treatment occurring during the pandemic [6–8]. Descriptions of the COVID-19 symptomatic experience among cancer patients are emerging. These patients are likely to be experiencing symptoms related to their cancer or its treatment before they develop COVID-19; many cancer-related symptoms, such as pain, fatigue, cognitive changes, and gastrointestinal symptoms, overlap with COVID-19 symptoms [7]. Common presenting symptoms in hospitalized cancer patients with acute COVID-19 include fever, cough, dyspnea, fatigue, myalgia, chest tightness, confusion, and headache [8, 9]. Little is known of the symptom burden of cancer patients with acute COVID-19 in the outpatient setting. In addition, patients with cancer are more likely than persons without cancer to become severely ill with COVID-19 and have increased risk for PASC [9–13]. From a public health perspective, empirically derived data, including data on symptom burden among persons with cancer and COVID-19, are needed to support predictive models of risk stratification for levels of care for both acute COVID-19 and PASC [6–8].

A valid and reliable patient-reported outcome (PRO) measure for capturing and quantifying the symptom burden related to COVID-19 was urgently needed as the pandemic evolved in spring 2020. In oncology care, accurate assessment of symptoms is critical to assist clinicians in identifying symptoms related to treatment toxicities, persistent cancer-related symptoms, acute COVID-19, and/or PASC to guide symptom-management [7]. Initial COVID-19 symptom checklists with yes/no, present/absent questions provided valuable early information and assisted with identifying patients who should be tested for COVID-19 [14–16]. However, quantified information directly reported by patients describing symptom burden of acute COVID-19 or PASC has been lacking [2, 7, 17]. Quarantine restrictions and isolation measures necessitated a method for patients to report symptoms and functional impairment remotely, directly, and routinely through electronic PROs. Electronic PROs can easily capture symptom evolution and trends over time, evaluate the patient’s response to treatment, and inform clinical care [18–20]. Importantly, a PRO that captures both the symptoms related to cancer and to COVID-19 can assist clinicians in assessing the spectrum of symptoms experienced by patients with diagnoses of cancer and COVID-19.

To that end, we aimed to rapidly develop, initially validate, and launch an electronic PRO measure of COVID-19 symptom burden, focused primarily on patients with cancer but that also may be useful for all patients with COVID-19.

## Methods

We collected data for this study under an MD Anderson Cancer Center Institutional Review Board (IRB)-approved protocol that allows collection of qualitative or quantitative symptom information from patients with cancer at MD Anderson Cancer Center. We selected the reliable, well-validated core MD Anderson Symptom Inventory (MDASI) as the base for the MDASI-COVID because our initial study population comprised patients with cancer and COVID-19.

### Measures

#### The MDASI-COVID

The core MDASI includes 13 symptom severity items common to most patients with cancer and 6 items of daily functioning with which symptoms may interfere [21]. Patients rate all items at their worst in the last 24 hours on 11-point scales ranging from 0 (symptom not present or no interference) to 10 (symptom as bad as can be imagined or complete interference) [21]. MDASI modules, such as the MDASI-COVID, contain all core MDASI items plus additional symptom items that are relevant to a particular disease or treatment.

We began in mid-March 2020 by conducting a web-based scan of US Centers for Disease Control (CDC) and World Health Organization (WHO) websites and publications to identify symptoms of COVID-19. An expert panel of 14 MD Anderson clinicians (representing pulmonary medicine, otolaryngology, critical care, and infectious diseases) caring for patients with COVID-19 reviewed and rated the relevance of the identified symptoms to patients with COVID-19 on a scale of 0 to 4 (0 = not relevant, 4 = very relevant). From these ratings, we developed a provisional MDASI-COVID comprising the core MDASI items plus additional symptom items with an expert-panel mean relevance rating of 3.0 or higher.

The MDASI-COVID is scored by calculating the mean value of the items within 2 scales (all symptom items and all interference items) and 4 subscales (the 13 core MDASI symptom items, the COVID-19–specific items, the physical interference items [walking, general activity, work; WAW], and the affective interference items [relations with others, enjoyment of life, mood; REM]). Patients must complete at least half of the items in a scale or subscale to be included in the analysis of that scale or subscale. All MDASI modules are scored in this manner.

#### EuroQOL Scales

The EuroQOL 5 Dimensions 5 Levels health status measure (EQ-5D-5L) comprises five dimensions (mobility, self-care, usual activities, pain/discomfort, anxiety/depression) graded on five levels (from no problem to an extreme problem) [22]. The digits for the five dimension ratings are combined into a 5-digit number representing the patient’s health state. This value is compared with population normative values to produce a utility index score [23].

The EQ visual analogue scale (EQ-VAS) records the patient’s self-rated health on a 0–100 vertical visual analogue scale with endpoints labeled “The worst health you can imagine” and “The best health you can imagine.” The EQ-VAS is a quantitative measure of the patient’s judgment of health status.

### Study participants and procedures

Starting in mid-May 2020 English-speaking adults who were ≥ 18 years of age, seeking care at MD Anderson but not employed at MD Anderson, and identified in the MD Anderson EPIC (Epic Systems Corporation, Verona, WI) electronic health record (EHR) as having a positive COVID-19 test were automatically sent an IRB-approved consent statement through their EPIC MyChart electronic patient portal. Patients who agreed to the consent statement by clicking an “agree to” button were automatically sent the provisional MDASI-COVID, the EQ-5D-5L, and the EQ-VAS to complete daily for the first 14 days after COVID-19 diagnosis, weekly from weeks 3 to 12 after diagnosis, and monthly from months 4 to 24 after diagnosis. Results of COVID-19 testing performed at MD Anderson were completed, reported, and automatically identified COVID-19 positive patients within approximately 12 hours. Patients not agreeing to the consent statement for multiple days after the consent statement was sent and/or having a positive COVID-19 test performed outside the institution and not identified as COVID-19 positive in the MD Anderson EPIC EHR for multiple days after diagnosis, received questionnaires to complete on the schedule based on the date that the positive COVID-19 sample was collected. Because we were interested ultimately, although not for the purposes of this report, in both acute COVID-19 and PASC symptoms, we allowed patients to begin study participation when they were able and willing.

The first assessment completed by each patient was used for the psychometric validation analysis of the MDASI-COVID. Outpatients reporting high levels (rated ≥ 7 on the MDASI-COVID’s 0–10 scale) of symptom severity that might require immediate attention (pain, shortness of breath, distress, sadness, fever or chills, chest heaviness or tightness, diarrhea) were flagged in the EHR and reported to the patients’ primary care teams electronically.

Because the MDASI-COVID, EQ-5D-5L, and EQ-VAS were built into the MyChart feature of the EPIC EHR, clinicians were able to view patient responses as soon as patients entered them. Through an institutional COVID-19 initiative (D3CODE), essential COVID-19 data elements (including sociodemographic data, clinical data, hospitalizations, and MDASI-COVID items) were automatically downloaded from EPIC into an institutional big-data database for use in research.

### Statistical analysis

The sample size was determined primarily on the basis of the MDASI-COVID’s ability to distinguish between patients hospitalized, including having a hospitalization extended, for COVID-19 versus those not hospitalized, as a measure of known-group validity. With 75 hospitalized patients and 523 patients not hospitalized, we would have 98% power to detect a half standard deviation difference in symptom severity between these two groups of patients, given a two-tailed test at a 5% significance level [24].

Sociodemographic and disease characteristics were analyzed descriptively. The prevalence and mean severity of symptoms were analyzed descriptively from the initial assessment. Hierarchical cluster analysis was used to better understand how the symptom items are interrelated, grouping symptoms that are perceived by patients to be similar in a progressive manner until all symptoms are included in a single hierarchy. Clusters were formed using Ward’s method with distances between symptoms calculated by using squared Euclidian distances and presented as a dendrogram.

The reliability of the MDASI-COVID was evaluated by calculating Cronbach alpha coefficients at baseline, as a measure of the internal consistency of responses to a group of items. The Cronbach alpha ranges from 0.0 to 1.0, with higher values indicating greater consistency and usually considered to be acceptable when Cronbach’s alpha is greater than or equal to 0.70 [25].

Test-retest reliability was evaluated by calculating Pearson correlation coefficients between the first assessment for each patient that was at least 21 days, but not more than 60 days, after diagnosis and a second assessment that was no more than 14 days after the first assessment. Known-group validity was established by using Student’s *t*-test to examine the differences between MDASI-COVID mean symptom severity and interference scale and subscale scores for hospitalized versus non-hospitalized patients. Patients who were hospitalized for COVID-19 were expected to have higher scale and subscale scores. Concurrent validity was tested by correlating the mean MDASI-COVID scale and subscale scores with the mean EQ-5D-5L utility index and EQ-VAS scores. Correlation values of 0.1, 0.3, and 0.5 were interpreted as small, medium, and large [25].

All *P* values reported are 2-tailed with a significance level of alpha < 0.05. All statistical procedures were performed by using SPSS statistical software for Windows (version 24, IBM SPSS, Inc., Chicago, IL).

## Results

### Development of the MDASI-COVID

The CDC/WHO web-based scan produced 31 symptoms of COVID-19; this list was ranked for relevance by the expert panel of clinicians. The initial MDASI-COVID included: the 13 core MDASI symptom items, of which 2 (fatigue, shortness of breath) had a mean rating ≥ 3.0 by the experts and 6 new symptom items identified from the literature review (coughing, fever/chills, malaise, chest heaviness/tightness, change in taste, change in sense of smell) rated ≥ 3.0 by the experts. In addition, we included 4 symptom items from the then-current CDC COVID-19 symptoms list (diarrhea, sore mouth/throat, muscle soreness/cramping, headache) [26] and 1 item deemed clinically relevant by the study team (muscle weakness). The 6 core MDASI interference items completed the MDASI-COVID (Table 1).

**Table 1:** Items Included in the Provisional MDASI-COVID

Source	Item
Core MDASI symptom items	Pain Fatigue^a^ Nausea Disturbed sleep Distress/feeling upset Shortness of breath^a^ Difficulty remembering Lack of appetite Drowsiness Dry mouth Sadness Vomiting Numbness/tingling
Initial COVID-specific symptom items	Chest heaviness/tightness^a^ Malaise^a^ Fever/chills^a^ Coughing^a^ Change in taste^a^ Change in sense of smell^a^ Diarrhea^b^ Muscle soreness/cramping^b^ Muscle weakness^c^ Sore mouth/throat^b^ Headache^b^
Additional COVID-specific symptom items added October 2020	Nasal congestion^d^ Eye problems^e^ Skin changes^e^
Core MDASI interference items [15]	General activity Mood Working (including housework) Relations with other people Walking Enjoyment of life

^a^ Rated ≥ 3.0 by the expert panel

^b^ US Centers for Disease Control COVID symptom list – April 2020

^c^ Study team recommendation

^d^ US Centers for Disease Control COVID symptom list – September 2020 [18]

^e^ Literature report

Abbreviations: COVID, novel coronavirus disease 2019; MDASI, MD Anderson Symptom Inventory

In October 2020, three additional symptoms (nasal congestion, eye problems, skin problems) were added to the provisional MDASI-COVID on the basis of the CDC’s revised COVID-19 symptom list. All items except change in sense of smell and nasal congestion were used in previous MDASI modules and have been cognitively debriefed with patients.

### Patient characteristics

Between May 15, 2020, and February 14, 2021, 2,154 patients were identified in the EPIC EHR as having tested positive for COVID-19 and were automatically sent an IRB-approved consent statement through MyChart. During the study period, 627 (29%) agreed to the consent statement; 27 of these patients were removed from the study because they did not meet eligibility criteria (did not speak English, were incorrectly identified as having a positive COVID-19 test, were < 18 years of age, or were MD Anderson employees). Another 209 patients (10%) declined the consent statement to complete the MDASI-COVID; the reasons they declined are unknown. The remaining 1,318 patients (61%) never responded to the MyChart message.

Sociodemographic and disease characteristics for the 600 participants are presented in Table 2. Of these, 598 completed the MDASI-COVID, EQ-5D-5L, and EQ-VAS at least once, and 361 completed the MDASI-COVID after the three new symptoms were added. Overall, patients averaged 4.7 (SD, 5.9) assessments and were an average of 35.5 days (SD, 47.7) from their first positive COVID-19 test at the initial assessment.

**Table 2:** Demographic and Clinical Characteristics of the Study Cohort (*N* = 600)

Characteristic	Mean (SD), range
Age, years	56.5 (14.1), 20–91
Time since COVID-19 diagnosis, days	35.7 (48.1), 1–271
	***n*** **(%)**
Sex	
Male	249 (41.5%)
Female	351 (58.5%)
Marital status	
Married or living with partner	437 (72.8%)
Single, divorced, or living alone	163 (27.2%)
Race	
White	517 (86.2%)
Black	63 (10.7%)
Asian	14 (2.3%)
Native Hawaiian or Pacific Islander	1 (0.2%)
American Indian or Alaskan Native	4 (0.7%)
Hispanic ethnicity	102 (17.0%)
Cancer diagnosis	
Solid tumor	483 (80.5%)
Hematological cancers	117 (19.5%)
History of hospitalization for COVID infection	75 (12.5%)
EQ-5D-5 L scores at baseline	
Utilities Index	0.82 (0.18), 0.02-1.00
VAS	78.3 (19.6), 0-100

Abbreviation: COVID-19, novel coronavirus disease 2019

### Symptom Severity and Prevalence

Table 3 presents the means of the MDASI-COVID symptom and interference items at initial assessment, rank-ordered from most severe to least severe. The percentages of participants reporting symptoms as not present, mild, moderate, or severe at initial assessment also are presented in Table 3. All symptom items were reported by at least 20% of respondents at the initial assessment.

**Table 3:** Descriptive Statistics for the MDASI-COVID Test Items

MDASICOVID	N	Mean	SD	Min/ Max	LCL	UCL	% = 0^a^	% 1–4^b^	% ≥5^c^	% ≥7^d^	% Missing^e^
Core symptoms (rank order)											
Fatigue	595	3.48	3.17	0/10	2.19	3.72	27.0	35.5	36.6	21.3	0.8
Drowsiness	596	2.49	2.87	0/10	2.26	2.74	39.0	36.3	24.0	14.0	0.7
Sleep disturbance	591	2.48	3.03	0/10	2.19	2.69	43.2	30.5	24.8	14.3	1.5
Distress	594	2.34	2.95	0/10	2.02	2.51	43.7	32.7	22.6	13.3	1.0
Pain	594	2.12	3.00	0/10	1.85	2.35	51.3	26.8	20.8	13.5	1.0
Lack of appetite	593	2.08	2.98	0/10	1.79	2.29	54.7	22.7	21.5	12.3	1.2
Dry mouth	594	2.07	2.92	0/10	1.80	2.29	51.5	28.5	20.0	11.8	1.0
Sadness	594	1.72	2.67	0/10	1.44	1.87	55.2	28.1	16.2	8.6	1.0
Difficulty remembering	596	1.61	2.45	0/10	1.37	1.78	55.8	29.0	14.6	7.0	0.7
Shortness of breath	594	1.56	2.56	0/10	1.30	1.73	60.0	23.3	15.7	7.7	1.0
Numbness	593	1.28	2.29	0/10	1.03	1.40	64.7	24.5	10.1	5.4	1.2
Nausea	594	1.05	2.22	0/10	0.88	1.25	71.8	17.2	10.0	4.7	1.0
Vomiting	595	0.54	1.83	0/10	0.38	0.67	64.7	24.2	10.0	5.3	1.2
Module items (rank order)											
Malaise	595	2.40	3.05	0/10	2.12	2.63	43.5	32.0	23.6	14.3	0.8
Change in taste	593	2.07	3.23	0/10	1.81	2.35	58.0	21.4	19.9	14.8	1.2
Muscle weakness	596	2.05	2.76	0/10	1.80	2.25	47.7	32.2	19.5	10.2	0.7
Change in smell	596	1.92	3.26	0/10	1.66	2.21	62.9	17.6	19.4	14.4	0.7
Muscle soreness	595	1.92	2.78	0/10	1.69	2.15	52.3	28.7	18.2	9.7	0.8
Headache	593	1.85	2.78	0/10	1.63	2.10	53.8	27.2	17.8	9.8	1.2
Nasal congestion	360	1.78	2.34	0/10	1.53	2.02	47.1	38.3	13.8	5.8	0.8
Coughing	595	1.62	2.38	0/10	1.40	1.80	50.0	36.5	12.7	6.5	0.8
Fever	596	1.44	2.71	0/10	1.21	1.66	67.7	17.6	14.2	8.7	0.7
Diarrhea	596	1.29	2.41	0/10	1.10	1.51	66.7	20.3	12.3	6.5	0.7
Chest heaviness	593	1.27	2.29	0/10	0.92	1.44	63.2	26.5	10.3	6.1	1.2
Eye problems	360	1.18	2.19	0/10	0.94	1.40	66.9	21.2	11.0	4.1	0.8
Sore mouth	594	1.10	2.18	0/10	0.92	1.29	68.2	22.3	8.5	5.2	1.0
Skin problems	358	0.85	1.91	0/10	0.63	1.01	74.7	17.6	6.4	3.6	1.4
Interference items (rank order)											
Work, including housework	596	2.91	3.47	0/10	2.63	3.19	44.2	25.2	30.0	20.3	0.7
General activity	595	2.72	3.27	0/10	2.40	2.95	43.0	28.2	28.0	17.0	0.8
Enjoyment of life	595	2.52	3.19	0/10	2.20	2.73	44.8	29.8	24.5	15.0	0.8
Mood	593	2.15	2.87	0/10	1.84	2.31	47.8	29.5	21.5	10.8	1.2
Walking	596	1.93	3.02	0/10	1.60	2.09	59.7	20.0	19.6	12.8	0.7
Relations with other people	592	1.86	2.91	0/10	1.58	2.06	57.2	23.2	18.3	9.8	1.3
Subscale scores											
13 core symptom items	597	1.91	1.99	0/9.31	1.73	2.05					
11 module items	597	1.72	2.06	0/10	1.32	1.87					
14 module items	597	1.65	1.96	0/9.29	1.47	1.86					
All 27 symptom items	598	1.79	1.90	0/9.30	1.62	1.93					
6 interference items	597	2.35	2.73	0/10	2.12	2.56					
WAW items	597	2.52	3.01	0/10	2.26	2.75					
REM items	597	2.18	2.68	0/10	1.95	2.38					

^a^ Percentage of patients who rated the item as not present (score = 0 on the 0–10 scale)

^b^ Percentage of patients who rated the item as mild (score = 1–4 on the 0–10 scale)

^c^ Percentage of patients who rated the item as moderate to severe (score = 5–10 on the 0–10 scale)

^d^ Percentage of patients who rated the item as severe (score = 7–10 on the 0–10 scale)

^e^ Percentage of patients who did not rate the item

Abbreviations: COVID, novel coronavirus disease 2019; LCL, lower 95% confidence limit; MDASI, MD Anderson Symptom Inventory; REM, composite of the interference items relations with other people, enjoyment of life, and mood; UCL, upper 95% confidence limit; WAW, composite of the interference items work, general activity, and walking

### Psychometric validation of the MDASI-COVID

#### Internal consistency reliability

Cronbach alphas for the 13 core items (0.927), 11 module items (0.923), 14 module items (0.924), all 24 symptom items (0.958), all 27 symptoms items (0.957), and interference items (0.937) at initial assessment indicate a high level of reliability across all scales.

#### Test-retest reliability

Most of the 103 assessments that met the criteria for test-retest reliability were for the original 24-symptom-item MDASI-COVID, so only the original 24 symptom items were used in the test-retest analysis. Mean time from diagnosis of COVID-19 to test assessment was 27.3 days (SD, 7.3 days) with a median of 25 days (range, 21–52 days). Mean time from diagnosis of COVID-19 to retest assessment was 34.3 days (SD, 7.7 days) with a median of 32 days (range, 23–58 days). Mean time between test and retest was 7.0 days (SD, 3.4 days) with a median of 7 days (range, 1–14 days). Pearson’s *r* for the 13 core symptom items was 0.772, for the 11 COVID-19-specific items was 0.702, for all 24 symptom items was 0.724, for the 6 interference items was 0.640, for the WAW interference items was 0.660, and for the REM interference items was 0.603.

#### Cluster analysis

**Fig. 1:**
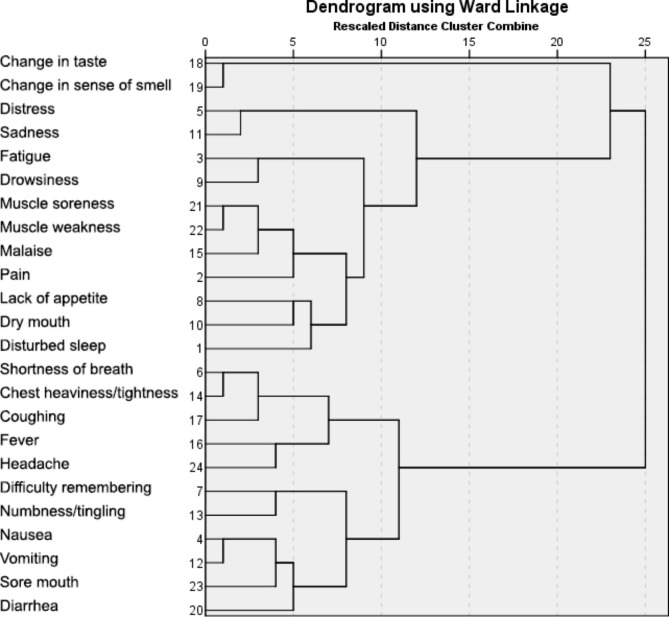
Dendrogram for Hierarchical Cluster Analysis of MDASI-COVID Core Symptom and Module Items (24 Items)

Figure 1 presents the hierarchical cluster analysis dendogram of the MDASI-COVID symptoms. The analysis showed seven clusters of related symptoms: sensory (change in taste and sense of smell), affective (distress and sadness), vitality (fatigue, drowsiness), constitutional (muscle soreness, muscle weakness, malaise, pain, lack of appetite, dry mouth, disturbed sleep), pulmonary (shortness of breath, chest heaviness or tightness, coughing, fever, headache), neurological (difficulty remembering, numbness or tingling), and gastrointestinal (nausea, vomiting, sore mouth, diarrhea). The affective, vitality, and constitutional clusters combined in a larger grouping, as did the pulmonary, neurological, and gastrointestinal clusters.

#### Known-group validity

Of the 598 patients who answered at least half of one or more of the scales or subscales (as required for inclusion in the known-group validity assessment), 75 (12.5%) were hospitalized for a diagnosis of COVID-19 and 523 (87.5%) were not. As predicted, there were significant differences in mean symptom severity and interference scales and subscales between the two groups at initial assessment (Table 4). The only subscale that did not show a significant difference between the two groups was the 11-item module subscale. The overall symptom severity (27 items) and interference (6 items) scales of the MDASI-COVID, as well as the symptom severity (13 items) and interference (6 items) scales of the MDASI Core, showed known group validity between patients who were hospitalized for a diagnosis of COVID-19 and those patients who were not.

**Table 4:** Known-Group Validity of the MDASI-COVID

MDASI-COVID subscale	Hospital status	*n*	Mean	SD	Mean difference	LCL of the diff 95%	UCL of the diff 95%	*P*	Cohen’s *d*
Core	Not hospitalized	522	1.81	1.95	–0.79	–1.27	-0.31	0.001*	0.40
	Hospitalized	75	2.61	2.12					
Module (11 items)	Not hospitalized	522	1.66	2.02	–0.48	–0.97	0.02	0.06	0.23
	Hospitalized	75	2.14	2.24					
Module (14 items)	Not hospitalized	522	1.59	1.92	–0.52	–0.99	–0.04	0.03*	0.26
	Hospitalized	75	2.111	2.19					
All symptom items (24 items)	Not hospitalized	523	1.74	1.90	–0.65	–1.12	–0.18	0.007*	0.33
	Hospitalized	75	2.39	2.11					
All symptom items (27 items)	Not hospitalized	523	1.70	1.86	–0.66	–1.12	–0.20	0.005*	0.35
	Hospitalized	75	2.36	2.08					
Interference	Not hospitalized	522	2.22	2.66	–1.10	–1.76	–0.33	0.001*	0.41
	Hospitalized	75	3.31	3.01					
WAW	Not hospitalized	522	2.35	2.93	–1.39	–2.11	–0.67	< 0.001*	0.47
	Hospitalized	75	3.74	3.28					
REM	Not hospitalized	522	2.08	2.63	–0.79	–1.44	-0.14	0.02*	0.30
	Hospitalized	75	2.87	2.98					

* Significant at *P* < .05

Abbreviations: COVID, novel coronavirus disease 2019; LCL, lower 95% confidence limit; MDASI, MD Anderson Symptom Inventory; REM, composite of the interference items relations with other people, enjoyment of life, and mood; UCL, upper 95% confidence limit; WAW, composite of the interference items work, general activity, and walking

#### Concurrent validity

All subscales of the MDASI-COVID and the EQ-5D-5 L utility index and EQ-VAS were significantly correlated (Table 5).

**Table 5:** Concurrent Validity of the MDASI-COVID Subscales

MDASI-COVID	Pearson correlation with the EQ-5D-5 L utility index	Pearson correlation with the EQVAS
Core	–0.674	–0.510
Module (11 Items)	–0.518	–0.403
Module (14 Items) ^a^	–0.531	–0.396
All symptoms (24 items)	–0.624	–0.470
All symptoms (27 items) ^a^	–0.616	–0.428
Interference	–0.699	–0.561
WAW	–0.695	–0.553
REM	–0.648	–0.526

Higher scores in the EQ-5D-5 L denote better outcome. All correlations were significant at *P* < .05

^a^ Subset of sample (n = 351 patients responding to 3 symptoms added in October 2020)

Abbreviations: COVID, novel coronavirus disease 2019; EQ-5D-5 L, EuroQOL 5 Dimensions 5 Levels; EQ-VAS, EQ-5D-5 L visual analog scale; MDASI, MD Anderson Symptom Inventory; REM, composite of the interference items relations with other people, enjoyment of life, and mood; WAW, composite of the interference items work, general activity, and walking

## Discussion

The MDASI-COVID is a PRO measure that contains a comprehensive set of symptoms commonly experienced by patients with cancer and COVID-19. The findings from this study confirm the initial psychometric validity (concurrent validity, known-group validity, test-retest reliability, and internal consistency) of the MDASI-COVID as a measure of symptom burden in patients with cancer and COVID-19 infection. Content validity for the MDASI-COVID was partially established through literature review and expert opinion; the instrument includes all symptoms in the current CDC COVID-19 symptom list, including more recent additions [2, 26–28]. Qualitative interviews with cancer patients who have had COVID-19 have been conducted and are being analyzed to verify that the content domain is valid and that no additional symptoms important to patients are missing.

The reliability coefficients for internal consistency reported here meet minimum requirements for acceptable reliability (α ≥ 0.7) [28]. The test-retest Pearson correlations also are minimally acceptable (*r* ≥ .6), especially considering the length of time between many of the tests [29, 30]. The MDASI-COVID showed known-group validity by discriminating between patients requiring hospitalization for COVID-19 versus not requiring hospitalization. The addition of the three symptom items of nasal congestion, eye problems, and skin problems resulted in the module symptom items differentiating significantly between hospitalized and non-hospitalized patients, demonstrating the relevance of these symptoms. Concurrent validity was shown through correlations between MDASI-COVID symptom and interference scales and subscales and EQ-5D-5L utility index scores. The EQ-5D-5L is a measure of patient health perception, a construct influenced by symptom and functional status (symptom burden) in the overall concept of health-related quality of life [31]. The significant correlations between the well-established EQ-5D-5L utility index and EQ-VAS scores and MDASI-COVID subscale scores support the validity of the MDASI-COVID as a measure of symptom burden specific to cancer patients with COVID-19.

We have shown that, in a health crisis, it is possible to quickly develop a provisional PRO measure that can be automated and implemented through an electronic patient portal to collect data on the patient experience of illness. Given the urgent need to gather PRO data early in the pandemic, rapid development of disease-specific measures was indicated. We used a systematic process to develop the measure, including literature review and expert panel review, before launching a provisional measure for use in clinical practice in the earliest phases of the COVID-19 pandemic. In turn, this data collected using the provisional measure can be used to determine the PRO measure’s initial validity, in support of the accuracy and reliability of the patient experience data. Patient-experience data collected electronically via an EHR portal can be easily combined with objective clinical data into big-data datasets for analysis. The D3CODE initiative, which provided the platform for collecting MDASI-COVID data, is an example of such an effort.

In our study we saw a large non-response rate to the initial request for participation in online survey data collection via the electronic health record (61%) and a large number of missing data from participants. Unfortunately, we were unable to track reasons for nonparticipation and non-response, but we had only 2 participants ask to be removed from the study. We hypothesize that patients who lacked internet access or who did not use the patient portal may not have received the invitation to participate and were involuntarily excluded. While we were able to quickly develop a provisional measure and provide the survey via automated messaging in the electronic patient portal, further investigation is needed explore the feasibility, acceptability, and usefulness of automated survey distribution via the electronic patient portal given the non-response rate and missing assessments.

The MDASI-COVID was designed to assess symptom prevalence, severity, and interference with functioning, [21, 32] whereas other COVID-19 symptom measures assess only symptom prevalence. Because the MDASI-COVID allows quantification of symptom severity and interference, it could be used to discern mild, moderate, and severe cases, which are substantially different in terms of recovery, illness impact, and health care utilization. The MDASI-COVID can be administered by paper and pencil, web-based patient portal, and mobile applications, providing flexibility and options for patients [33]. The MDASI-COVID is comprehensive but brief enough to avoid being burdensome to patients and clinicians [33].

Both the previously validated MDASI Core [21] and the MDASI-COVID were able to demonstrate a higher symptom burden in patients who required hospitalization for a diagnosis of COVID-19 than in patients who did not require hospitalization because of a diagnosis of COVID-19. Although designed to measure cancer symptom burden, many of the symptoms included in the MDASI Core are common in patients with COVID-19 [7]. Both the MDASI Core and the MDASI-COVID can be used to monitor symptom burden in patients with COVID-19. If a shorter questionnaire is desired due to frequency of administration or severe debility of patients, the MDASI Core may be preferable. However, the MDASI-COVID includes additional COVID-specific symptom items that may be of use to both clinicians and in research.

Longitudinal assessment with the MDASI-COVID in patients with cancer is ongoing to provide additional evidence of its sensitivity to clinical changes and its ability to predict patient outcomes. The results of this study will provide a description of the trajectory of symptom burden of acute COVID-19 and PASC in cancer patients with COVID-19. A separate study in patients with COVID-19 but without cancer is underway to determine the MDASI-COVID’s validity and usefulness in this population. Finally, qualitative interviews with patients who have had COVID-19 are being analyzed to confirm the content validity of the MDASI-COVID.

As a brief measure of the symptom burden of COVID-19 in patients with cancer, the MDASI-COVID provides clinicians with easily interpreted symptom severity scores and interference with daily functioning scores that can be rapidly and frequently assessed and acted upon in clinical care. The measure may be useful for oncology clinicians monitoring patients with both cancer and COVID-19, where the MDASI core symptoms may not sufficiently capture all symptoms unique to the COVID-19 experience. Going forward, inclusion of PROs in the care of patients with PASC may be useful for characterizing the net clinical benefit of treatment, given the substantial and emerging symptom burden in this patient population. This is especially important, in that no current guidelines exist for how to assess and manage post-COVID patients.

### Study Limitations

Study participants were recruited by using the EHR patient portal of a single comprehensive cancer center in the United States. Thus, patients who lacked internet access or did not use the patient portal were involuntarily excluded from participation. In addition, because we were unable to track reasons for nonparticipation, we cannot determine whether patients with more severe symptoms or poorer performance status were less likely to participate and provide symptom data.

Not all patients began study participation immediately after a positive COVID-19 test result. Therefore, study results are more likely to reflect symptoms experienced later in the course of COVID-19 (i.e., 30 days or more after diagnosis rather than early acute symptoms). Further analysis of the existing data is needed to describe the symptomatic trajectory of COVID-19. Our data set will allow us to do this for the variants of COVID-19 that were prevalent through 2020 and early 2021.

As the COVID-19 pandemic has progressed, variants have emerged, people have developed immunity by vaccination or infection, and treatments have improved, causing the symptoms of COVID-19 to evolve. Many of the symptoms included in the MDASI-COVID are still reported as common to patients with COVID-19. However, the incidence and severity of the symptoms may have changed. Additional research with the MDASI-COVID is needed as the disease evolves.

## Conclusion

The MDASI-COVID is a concise measure of the symptom burden of patients with cancer and COVID-19 that has preliminary validity and reliability for use in clinical care and in research. The MDASI-COVID may be useful for tracking commonly experienced symptoms and assessing change in symptom severity over time for patients with acute COVID-19 or PASC. Additional research is needed to confirm the content domain and content validity of the MDASI-COVID among patients who are not diagnosed with cancer and among patients with PASC. Exploration of the factor structure of the questionnaire and establishment of additional validity, such as predictive validity for identifying patients who may require higher levels of care, is needed. Longitudinal measurement and correlation with clinical factors will confirm the sensitivity of the questionnaire.

A provisional PRO can be rapidly developed and implemented through the patient portal of an EHR to collect data on the patient experience of disease during a large, unexpected public health crisis. Data on the patient experience of disease and its sequelae in a public health crisis can inform care, suggest long-term resources that will be needed, drive public policy decisions, and contribute to larger data analyses that will be vital to understanding how to respond to crises.

## Electronic supplementary material

Below is the link to the electronic supplementary material.

Supplementary Material 1

## Data Availability

The datasets used and/or analyzed during the current study are available from the corresponding author on reasonable request.

## List of Abbreviations

COVID-19: SARS-CoV-2 infection
MDASI-COVID: MD Anderson Symptom Inventory for COVID-19
PRO: Patient-reported outcome
PASC: Persistent postacute sequelae of COVID-19
SARS-CoV-2: Severe acute respiratory syndrome-associated coronavirus 2
