# Supplementary material for: Measuring symptom burden in patients with cancer during a pandemic: the MD Anderson symptom inventory for COVID-19 (MDASI-COVID)
Source: J Patient Rep Outcomes. 2023 May 26;7:48. doi: 10.1186/s41687-023-00591-x (PMC10215036; doi:10.1186/s41687-023-00591-x)
Supplement: Supplementary file 1 — Supplementary Material 1 [file 41687_2023_591_MOESM1_ESM.docx]

# Measuring Symptom Burden in Patients with Cancer During a Pandemic: The MD Anderson Symptom Inventory for Covid-19 (MDASI-COVID)

# ABSTRACT

**BACKGROUND:** Symptom expression in SARS-CoV-2 infection (COVID-19) may affect patients already symptomatic with cancer. Patient-reported outcomes (PROs) can describe symptom burden during the acute and postacute stages of COVID-19 and support risk stratification for levels of care. At the start of the COVID-19 pandemic, our purpose was to rapidly develop, launch through an electronic patient portal, and provide initial validation for a PRO measure of COVID-19 symptom burden in patients with cancer.

**METHODS:** We conducted a CDC/WHO web-based scan for COVID-19 symptoms and a relevance review of symptoms by an expert panel of clinicians treating cancer patients with COVID-19 to create a provisional MD Anderson Symptom Inventory for COVID-19 (MDASI-COVID). English-speaking adults with cancer who tested positive for COVID-19 participated in the psychometric testing phase. Patients completed longitudinal assessments of the MDASI-COVID and the EuroQOL 5 Dimensions 5 Levels (EQ-5D-5L) utility index and visual analog scale, which were presented through an electronic health record patient portal. To test the validity of the MDASI-COVID to distinguish between known groups of patients, we hypothesized that patients hospitalized, including having a hospitalization extended, for COVID-19 versus those not hospitalized would experience higher symptom burden. Correlation of mean symptom severity and interference scores with relevant EQ-5D-5L scores tested concurrent validity. The reliability of the MDASI-COVID was evaluated by calculating Cronbach alpha coefficients and test-retest reliability was evaluated by calculating Pearson correlation coefficients between the initial assessment and a second assessment no more than 14 days later.

**RESULTS:** The web-based scan found 31 COVID-19‒related symptoms; rankings of a 14-clinician expert panel reduced this list to 11 COVID-specific items to be added to the core MDASI. Time from literature scan start in March 2020 to instrument launch in May 2020 was 2 months. Psychometric analysis established the MDASI-COVID’s reliability, known-group validity, and concurrent validity.

**CONCLUSIONS:** We were able to rapidly develop and electronically launch a PRO measure of COVID-19 symptom burden in patients with cancer. Additional research is needed to confirm the content domain and predictive validity of the MDASI-COVID and define the symptom burden trajectory of COVID-19.

**KEYWORDS:** COVID-19; electronic patient-reported outcomes; symptoms; symptom burden; cancer; quality of life

# background

Understanding of the symptom burden associated with the viral infection caused by severe acute respiratory syndrome-associated coronavirus 2 (SARS-CoV-2, also known as COVID-19) continues to evolve. Symptom expression in COVID-19 ranges from asymptomatic to extremely severe and from acute symptoms to symptoms related to persistent postacute sequelae of COVID-19 (PASC; also called long COVID). Prolonged symptom burden and morbidity related to COVID-19 infection are increasingly apparent, especially among adults hospitalized with severe COVID-19. ^1^

Common acute symptoms of COVID-19 include fever, cough, myalgia, fatigue, shortness of breath, joint pain, headache, gastrointestinal symptoms, and altered sense of smell and taste. ^2,3^ Approximately one-third of adults treated for COVID-19 in the outpatient setting reported that they had not returned to usual health within 2–3 weeks of testing positive, ^1^ while 87% of patients recovered from COVID-19 had one or more persistent symptoms, commonly fatigue and shortness of breath. ^3^ Symptoms found more frequently in PASC than in other severe illnesses include loss of smell and taste, memory loss, chest pain, difficulty concentrating, confusion, and bone and joint pain. ^4^ There is no current consensus on the specific symptoms involved in PASC due to its variable, multisystem nature. ^5^

Understanding the symptom burden and related health impact of COVID-19 is important for patients with cancer given that this population is vulnerable to the direct impacts of COVID-19 infection and to delays in cancer diagnosis and treatment occurring during the pandemic. ^6-8^ Descriptions of the COVID-19 symptomatic experience among cancer patients are emerging. These patients are likely to be experiencing symptoms related to their cancer or its treatment before they develop COVID-19; many cancer-related symptoms, such as pain, fatigue, cognitive changes, and gastrointestinal symptoms, overlap with COVID-19 symptoms. ^7^ Common presenting symptoms in hospitalized cancer patients with acute COVID-19 include fever, cough, dyspnea, fatigue, myalgia, chest tightness, confusion, and headache. ^8-9^ Little is known of the symptom burden of cancer patients with acute COVID-19 in the outpatient setting. In addition, patients with cancer are more likely than persons without cancer to become severely ill with COVID-19 and have increased risk for PASC. ^9-13^ From a public health perspective, empirically derived data, including data on symptom burden among persons with cancer and COVID-19, are needed to support predictive models of risk stratification for levels of care for both acute COVID-19 and PASC. ^6-8^

A valid and reliable patient-reported outcome (PRO) measure for capturing and quantifying the symptom burden related to COVID-19 was urgently needed as the pandemic evolved in spring 2020. In oncology care, accurate assessment of symptoms is critical to assist clinicians in identifying symptoms related to treatment toxicities, persistent cancer-related symptoms, acute COVID-19, and/or PASC to guide symptom-management. ^7^ Initial COVID-19 symptom checklists with yes/no, present/absent questions provided valuable early information and assisted with identifying patients who should be tested for COVID-19. ^14-16^ However, quantified information directly reported by patients describing symptom burden of acute COVID-19 or PASC has been lacking. ^2,7,17^ Quarantine restrictions and isolation measures necessitated a method for patients to report symptoms and functional impairment remotely, directly, and routinely through electronic PROs. Electronic PROs can easily capture symptom evolution and trends over time, evaluate the patient’s response to treatment, and inform clinical care. ^18-20^ Importantly, a PRO that captures both the symptoms related to cancer and to COVID-19 can assist clinicians in assessing the spectrum of symptoms experienced by patients with diagnoses of cancer and COVID-19.

To that end, we aimed to rapidly develop, initially validate, and launch an electronic PRO measure of COVID-19 symptom burden, focused primarily on patients with cancer but also may be useful for all patients with COVID-19.

# METHODS

We collected data for this study under an MD Anderson Cancer Center Institutional Review Board (IRB)-approved protocol that allows collection of qualitative or quantitative symptom information from patients with cancer at MD Anderson Cancer Center. We selected the reliable, well-validated core MD Anderson Symptom Inventory (MDASI) as the base for the MDASI-COVID because our initial study population comprised patients with cancer and COVID-19.

## Measures

### The MDASI-COVID

The core MDASI includes 13 symptom severity items common to most patients with cancer and 6 items of daily functioning with which symptoms may interfere. ^21^ Patients rate all items at their worst in the last 24 hours on 11-point scales ranging from 0 (symptom not present or no interference) to 10 (symptom as bad as can be imagined or complete interference). ^21^ MDASI modules, such as the MDASI-COVID, contain all core MDASI items plus additional symptom items that are relevant to a particular disease or treatment.

We began in mid-March 2020 by conducting a web-based scan of US Centers for Disease Control (CDC) and World Health Organization (WHO) websites and publications to identify symptoms of COVID-19. An expert panel of 14 MD Anderson clinicians (representing pulmonary medicine, otolaryngology, critical care, and infectious diseases) caring for patients with COVID-19 reviewed and rated the relevance of the identified symptoms to patients with COVID-19 on a scale of 0 to 4 (0=not relevant, 4=very relevant). From these ratings, we developed a provisional MDASI-COVID comprising the core MDASI items plus additional symptom items with an expert-panel mean relevance rating of 3.0 or higher.

The MDASI-COVID is scored by calculating the mean value of the items within 2 scales (all symptom items and all interference items) and 4 subscales (the 13 core MDASI symptom items, the COVID-19–specific items, the physical interference items [walking, general activity, work; WAW], and the affective interference items [relations with others, enjoyment of life, mood; REM]). Patients must complete at least half of the items in a scale or subscale to be included in the analysis of that scale or subscale. All MDASI modules are scored in this manner.

### EuroQOL Scales

The EuroQOL 5 Dimensions 5 Levels health status measure (EQ-5D-5L) comprises five dimensions (mobility, self-care, usual activities, pain/discomfort, anxiety/depression) graded on five levels (from no problem to an extreme problem). ^22^ The digits for the five dimension ratings are combined into a 5-digit number representing the patient’s health state. This value is compared with population normative values to produce a utility index score.^23^

The EQ visual analogue scale (EQ-VAS) records the patient’s self-rated health on a 0–100 vertical visual analogue scale with endpoints labeled “The worst health you can imagine” and “The best health you can imagine.” The EQ-VAS is a quantitative measure of the patient’s judgment of health status.

## Study Participants and Procedures

Starting in mid-May 2020 English-speaking adults who were ≥18 years of age, seeking care at MD Anderson but not employed at MD Anderson, and identified in the MD Anderson EPIC (Epic Systems Corporation, Verona, WI) electronic health record (EHR) as having a positive COVID-19 test were automatically sent an IRB-approved consent statement through their EPIC MyChart electronic patient portal. Patients who agreed to the consent statement by clicking an “agree to” button were automatically sent the provisional MDASI-COVID, the EQ-5D-5L, and the EQ-VAS to complete daily for the first 14 days after COVID-19 diagnosis, weekly from weeks 3 to 12 after diagnosis, and monthly from months 4 to 24 after diagnosis. Results of COVID-19 testing performed at MD Anderson were completed, reported, and automatically identified COVID-19 positive patients within approximately 12 hours. Patients not agreeing to the consent statement for multiple days after the consent statement was sent and/or having a positive COVID-19 test performed outside the institution and not identified as COVID-19 positive in the MD Anderson EPIC EHR for multiple days after diagnosis, received questionnaires to complete on the schedule based on the date that the positive COVID-19 sample was collected. Because we were interested ultimately, although not for the purposes of this report, in both acute COVID-19 and PASC symptoms, we allowed patients to begin study participation when they were able and willing.

The first assessment completed by each patient was used for the psychometric validation analysis of the MDASI-COVID. Outpatients reporting high levels (rated ≥7 on the MDASI-COVID’s 0–10 scale) of symptom severity that might require immediate attention (pain, shortness of breath, distress, sadness, fever or chills, chest heaviness or tightness, diarrhea) were flagged in the EHR and reported to the patients’ primary care teams electronically.

Because the MDASI-COVID, EQ-5D-5L, and EQ-VAS were built into the MyChart feature of the EPIC EHR, clinicians were able to view patient responses as soon as patients entered them. Through an institutional COVID-19 initiative (D3CODE), essential COVID-19 data elements (including sociodemographic data, clinical data, hospitalizations, and MDASI-COVID items) were automatically downloaded from EPIC into an institutional big-data database for use in research.

## Statistical Analysis

The sample size was determined primarily on the basis of the MDASI-COVID’s ability to distinguish between patients hospitalized, including having a hospitalization extended, for COVID-19 versus those not hospitalized, as a measure of known-group validity. With 75 hospitalized patients and 524 patients not hospitalized, we would have 98% power to detect a half standard deviation difference in symptom severity between these two groups of patients, given a two-tailed test at a 5% significance level. ^24^

Sociodemographic and disease characteristics were analyzed descriptively. The prevalence and mean severity of symptoms were analyzed descriptively from the initial assessment. Hierarchical cluster analysis was used to better understand how the symptom items are interrelated, grouping symptoms that are perceived by patients to be similar in a progressive manner until all symptoms are included in a single hierarchy. Clusters were formed using Ward’s method with distances between symptoms calculated by using squared Euclidian distances and presented as a dendrogram.

The reliability of the MDASI-COVID was evaluated by calculating Cronbach alpha coefficients at baseline, as a measure of the internal consistency of responses to a group of items. The Cronbach alpha ranges from 0.0 to 1.0, with higher values indicating greater consistency and usually considered to be acceptable when Cronbach’s alpha is greater than or equal to 0.70. ^25^

Test-retest reliability was evaluated by calculating Pearson correlation coefficients between the first assessment for each patient that was at least 21 days, but not more than 60 days, after diagnosis and a second assessment that was no more than 14 days after the first assessment. Known-group validity was established by using Student’s *t*-test to examine the differences between MDASI-COVID mean symptom severity and interference scale and subscale scores for hospitalized versus non-hospitalized patients. Patients who were hospitalized for COVID-19 were expected to have higher scale and subscale scores. Concurrent validity was tested by correlating the mean MDASI-COVID scale and subscale scores with the mean EQ-5D-5L utility index and EQ-VAS scores. Correlation values of 0.1, 0.3, and 0.5 were interpreted as small, medium, and large. ^25^

All *P* values reported are 2-tailed with a significance level of alpha < 0.05. All statistical procedures were performed by using SPSS statistical software for Windows (version 24, IBM SPSS, Inc., Chicago, IL).

# rESULTS

## Development of the MDASI-COVID

The CDC/WHO web-based scan produced 31 symptoms of COVID-19; this list was ranked for relevance by the expert panel of clinicians. The initial MDASI-COVID included: the 13 core MDASI symptom items, of which 2 (fatigue, shortness of breath) had a mean rating >3.0 by the experts and 6 new symptom items identified from the literature review (coughing, fever/chills, malaise, chest heaviness/tightness, change in taste, change in sense of smell) rated >3.0 by the experts. In addition, we included 4 symptom items from the then-current CDC COVID-19 symptoms list (diarrhea, sore mouth/throat, muscle soreness/cramping, headache) ^26^ and 1 item deemed clinically relevant by the study team (muscle weakness. The 6 core MDASI interference items completed the MDASI-COVID (Table 1).

In October 2020, three additional symptoms (nasal congestion, eye problems, skin problems) were added to the provisional MDASI-COVID on the basis of the CDC’s revised COVID-19 symptom list. All items except change in sense of smell and nasal congestion were used in previous MDASI modules and have been cognitively debriefed with patients.

## Patient Characteristics

Between May 15, 2020, and February 14, 2021, 2,154 patients were identified in the EPIC EHR as having tested positive for COVID-19 and were automatically sent an IRB-approved consent statement through MyChart. During the study period, 627 (29%) agreed to the consent statement; 27 of these patients were removed from the study because they did not meet eligibility criteria (did not speak English, were incorrectly identified as having a positive COVID-19 test, were <18 years of age, or were MD Anderson employees). Another 209 patients (10%) declined the consent statement to complete the MDASI-COVID; the reasons they declined are unknown. The remaining 1,318 patients (61%) never responded to the MyChart message.

Sociodemographic and disease characteristics for the 600 participants are presented in Table 2. Of these, 598 completed the MDASI-COVID, EQ-5D-5L, and EQ-VAS at least once, and 361 completed the MDASI-COVID after the three new symptoms were added. Overall, patients averaged 4.7 (SD, 5.9) assessments and were an average of 35.5 days (SD, 47.7) from their first positive COVID-19 test at the initial assessment.

## Symptom Severity and Prevalence

Table 3 presents the means of the MDASI-COVID symptom and interference items at initial assessment, rank-ordered from most severe to least severe. The percentages of participants reporting symptoms as not present, mild, moderate, or severe at initial assessment also are presented in Table 3. All symptom items were reported by at least 20% of respondents at the initial assessment.

## Psychometric Validation of the MDASI-COVID

### Internal Consistency Reliability

Cronbach alphas for the 13 core items (0.927), 11 module items (0.923), 14 module items (0.924), all 24 symptom items (0.958), all 27 symptoms items (0.957), and interference items (0.937) at initial assessment indicate a high level of reliability across all scales.

### Test-Retest Reliability

Most of the 103 assessments that met the criteria for test-retest reliability were for the original 24-symptom-item MDASI-COVID, so only the original 24 symptom items were used in the test-retest analysis. Mean time from diagnosis of COVID-19 to test assessment was 27.3 days (SD, 7.3 days) with a median of 25 days (range, 21–52 days). Mean time from diagnosis of COVID-19 to retest assessment was 34.3 days (SD, 7.7 days) with a median of 32 days (range, 23–58 days). Mean time between test and retest was 7.0 days (SD, 3.4 days) with a median of 7 days (range, 1–14 days). Pearson’s *r* for the 13 core symptom items was 0.772, for the 11 COVID-19-specific items was 0.702, for all 24 symptom items was 0.724, for the 6 interference items was 0.640, for the WAW interference items was 0.660, and for the REM interference items was 0.603.

### Cluster Analysis

Figure 1 presents the hierarchical cluster analysis dendogram of the MDASI-COVID symptoms. The analysis showed seven clusters of related symptoms: sensory (change in taste and sense of smell), affective (distress and sadness), vitality (fatigue, drowsiness), constitutional (muscle soreness, muscle weakness, malaise, pain, lack of appetite, dry mouth, disturbed sleep), pulmonary (shortness of breath, chest heaviness or tightness, coughing, fever, headache), neurological (difficulty remembering, numbness or tingling), and gastrointestinal (nausea, vomiting, sore mouth, diarrhea). The affective, vitality, and constitutional clusters combined in a larger grouping, as did the pulmonary, neurological, and gastrointestinal clusters.

### Known-Group Validity

Of the 598 patients who answered at least half of one or more of the scales or subscales (as required for inclusion in the known-group validity assessment), 75 (12.5%) were hospitalized for a diagnosis of COVID-19 and 523 (87.5%) were not. As predicted, there were significant differences in mean symptom severity and interference scales and subscales between the two groups at initial assessment (Table 4). The only subscale that did not show a significant difference between the two groups was the 11-item module subscale. The overall symptom severity (27 items) and interference (6 items) scales of the MDASI-COVID, as well as the symptom severity (13 items) and interference (6 items) scales of the MDASI Core, showed known group validity between patients who were hospitalized for a diagnosis of COVID-19 and those patients who were not.

### Concurrent Validity

All subscales of the MDASI-COVID and the EQ-5D-5L utility index and EQ-VAS were significantly correlated (Table 5).

# Discussion

The MDASI-COVID is a PRO measure that contains a comprehensive set of symptoms commonly experienced by patients with cancer and COVID-19. The findings from this study confirm the initial psychometric validity (concurrent validity, known-group validity, test-retest reliability, and internal consistency) of the MDASI-COVID as a measure of symptom burden in patients with cancer and COVID-19 infection. Content validity for the MDASI-COVID was partially established through literature review and expert opinion; the instrument includes all symptoms in the current CDC COVID-19 symptom list, including more recent additions. ^2,26-28^ Qualitative interviews with cancer patients who have had COVID-19 have been conducted and are being analyzed to verify that the content domain is valid and that no additional symptoms important to patients are missing.

The reliability coefficients for internal consistency reported here meet minimum requirements for acceptable reliability (α ≥ 0.7). ^28^ The test-retest Pearson correlations also are minimally acceptable (*r* ≥ 0.6), especially considering the length of time between many of the tests. ^29^ The MDASI-COVID showed known-group validity by discriminating between patients requiring hospitalization for COVID-19 versus not requiring hospitalization. The addition of the three symptom items of nasal congestion, eye problems, and skin problems resulted in the module symptom items differentiating significantly between hospitalized and non-hospitalized patients, demonstrating the relevance of these symptoms. Concurrent validity was shown through correlations between MDASI-COVID symptom and interference scales and subscales and EQ-5D-5L utility index scores. The EQ-5D-5L is a measure of patient health perception, a construct influenced by symptom and functional status (symptom burden) in the overall concept of health-related quality of life. ^31^ The significant correlations between the well-established EQ-5D-5L utility index and EQ-VAS scores and MDASI-COVID subscale scores support the validity of the MDASI-COVID as a measure of symptom burden specific to cancer patients with COVID-19.

We have shown that, in a health crisis, it is possible to quickly develop a provisional PRO measure that can be automated and implemented through an electronic patient portal to collect data on the patient experience of illness. Given the urgent need to gather PRO data early in the pandemic, rapid development of disease-specific measures was indicated. We used a systematic process to develop the measure, including literature review and expert panel review, before launching a provisional measure for use in clinical practice in the earliest phases of the COVID-19 pandemic. In turn, this data collected using the provisional measure can be used to determine the PRO measure’s initial validity, in support of the accuracy and reliability of the patient experience data. Patient-experience data collected electronically via an EHR portal can be easily combined with objective clinical data into big-data datasets for analysis. The D3CODE initiative, which provided the platform for collecting MDASI-COVID data, is an example of such an effort.

In our study we saw a large non-response rate to the initial request for participation in online survey data collection via the electronic health record (61%) and a large number of missing data from participants. Unfortunately, we were unable to track reasons for nonparticipation and non-response, but we had only 2 participants ask to be removed from the study. We hypothesize that patients who lacked internet access or who did not use the patient portal may not have received the invitation to participate and were involuntarily excluded. While we were able to quickly develop a provisional measure and provide the survey via automated messaging in the electronic patient portal, further investigation is needed explore the feasibility, acceptability, and usefulness of automated survey distribution via the electronic patient portal given the non-response rate and missing assessments.

The MDASI-COVID was designed to assess symptom prevalence, severity, and interference with functioning, ^21,32^ whereas other COVID-19 symptom measures assess only symptom prevalence. Because the MDASI-COVID allows quantification of symptom severity and interference, it could be used to discern mild, moderate, and severe cases, which are substantially different in terms of recovery, illness impact, and health care utilization. The MDASI-COVID can be administered by paper and pencil, web-based patient portal, and mobile applications, providing flexibility and options for patients. ^33^ The MDASI-COVID is comprehensive but brief enough to avoid being burdensome to patients and clinicians. ^33^

Both the previously validated MDASI Core^21^ and the MDASI-COVID were able to demonstrate a higher symptom burden in patients who required hospitalization for a diagnosis of COVID-19 than in patients who did not require hospitalization because of a diagnosis of COVID-19. Although designed to measure cancer symptom burden, many of the symptoms included in the MDASI Core are common in patients with COVID-19.^7^ Both the MDASI Core and the MDASI-COVID can be used to monitor symptom burden in patients with COVID-19. If a shorter questionnaire is desired due to frequency of administration or severe debility of patients, the MDASI Core may be preferable. However, the MDASI-COVID includes additional COVID-specific symptom items that may be of use to both clinicians and in research.

Longitudinal assessment with the MDASI-COVID in patients with cancer is ongoing to provide additional evidence of its sensitivity to clinical changes and its ability to predict patient outcomes. The results of this study will provide a description of the trajectory of symptom burden of acute COVID-19 and PASC in cancer patients with COVID-19. A separate study in patients with COVID-19 but without cancer is underway to determine the MDASI-COVID’s validity and usefulness in this population. Finally, qualitative interviews with patients who have had COVID-19 are being analyzed to confirm the content validity of the MDASI-COVID.

As a brief measure of the symptom burden of COVID-19 in patients with cancer, the MDASI-COVID provides clinicians with easily interpreted symptom severity scores and interference with daily functioning scores that can be rapidly and frequently assessed and acted upon in clinical care. The measure may be useful for oncology clinicians monitoring patients with both cancer and COVID-19, where the MDASI core symptoms may not sufficiently capture all symptoms unique to the COVID-19 experience. Going forward, inclusion of PROs in the care of patients with PASC may be useful for characterizing the net clinical benefit of treatment, given the substantial and emerging symptom burden in this patient population. This is especially important, in that no current guidelines exist for how to assess and manage post-COVID patients.

## Study Limitations

Study participants were recruited by using the EHR patient portal of a single comprehensive cancer center in the United States. Thus, patients who lacked internet access or did not use the patient portal were involuntarily excluded from participation. In addition, because we were unable to track reasons for nonparticipation, we cannot determine whether patients with more severe symptoms or poorer performance status were less likely to participate and provide symptom data.

Not all patients began study participation immediately after a positive COVID-19 test result. Therefore, study results are more likely to reflect symptoms experienced later in the course of COVID-19 (i.e., 30 days or more after diagnosis rather than early acute symptoms). Further analysis of the existing data is needed to describe the symptomatic trajectory of COVID-19. Our data set will allow us to do this for the variants of COVID-19 that were prevalent through 2020 and early 2021.

As the COVID-19 pandemic has progressed, variants have emerged, people have developed immunity by vaccination or infection, and treatments have improved, causing the symptoms of COVID-19 to evolve. Many of the symptoms included in the MDASI-COVID are still reported as common to patients with COVID-19. However, the incidence and severity of the symptoms may have changed. Additional research with the MDASI-COVID is needed as the disease evolves.

## Conclusion

The MDASI-COVID is a concise measure of the symptom burden of patients with cancer and COVID-19 that has preliminary validity and reliability for use in clinical care and in research. The MDASI-COVID may be useful for tracking commonly experienced symptoms and assessing change in symptom severity over time for patients with acute COVID-19 or PASC. Additional research is needed to confirm the content domain and content validity of the MDASI-COVID among patients who are not diagnosed with cancer and among patients with PASC. Exploration of the factor structure of the questionnaire and establishment of additional validity, such as predictive validity for identifying patients who may require higher levels of care, is needed. Longitudinal measurement and correlation with clinical factors will confirm the sensitivity of the questionnaire.

A provisional PRO can be rapidly developed and implemented through the patient portal of an EHR to collect data on the patient experience of disease during a large, unexpected public health crisis. Data on the patient experience of disease and its sequelae in a public health crisis can inform care, suggest long-term resources that will be needed, drive public policy decisions, and contribute to larger data analyses that will be vital to understanding how to respond to crises.

**List of Abbreviations**

COVID-19, SARS-CoV-2 infection

MDASI-COVID, MD Anderson Symptom Inventory for COVID-19

PRO, patient-reported outcome

PASC, persistent postacute sequelae of COVID-19

SARS-CoV-2, severe acute respiratory syndrome-associated coronavirus 2

# References

1. Tenforde MW, Kim SS, Lindsell CJ et al. (2020) Symptom duration and risk factors for delayed return to usual health among outpatients with COVID-19 in a multistate health care systems network - United States, March-June 2020. MMWR Morb Mortal Wkly Rep 69(30):993-998. doi:10.15585/mmwr.mm6930e1.

2. Huang C, Wang Y, Li X et al. (2020) Clinical features of patients infected with 2019 novel coronavirus in Wuhan, China. Lancet 395(10223):497-506. doi:10.1016/S0140-6736(20)30183-5.

3. Carfì A, Bernabei R, Landi F (2020) Gemelli Against Covid-Post-Acute Care Study Group. Persistent symptoms in patients after acute COVID-19. JAMA 324(6):603-605. doi:10.1001/jama.2020.12603.

4. Rando HM, Bennett TD, Byrd JB et al. (2021) Challenges in defining long COVID: striking differences across literature, electronic health records, and patient-reported information. medRxiv. doi:10.1101/2021.03.20.21253896

5. Oronsky B, Larson C, Hammond TC et al. (2021) A review of persistent post-COVID syndrome (PPCS). Clin Rev Allergy Immunol. doi:10.1007/s12016-021-08848-3

6. Weerahandi H, Hochman KA, Simon E et al. (2020) Post-discharge health status and symptoms in patients with severe COVID-19. medRxiv. doi:10.1101/2020.08.11.20172742

7. Miaskowski C, Paul SM, Snowberg K et al. (2021) Oncology patients' perceptions of and experiences with COVID-19. Support Care Cancer 29(4):1941-1950. doi: 10.1007/s00520-020-05684-7.

8. Zarifkar P, Kamath A, Robinson C et al. (2021) Clinical characteristics and outcomes in patients with covid-19 and cancer: a systematic review and meta-analysis. Clin Onc 33(3), e180–e191. doi: https://doi.org/10.1016/j.clon.2020.11.006.

9. Joharatnam-Hogan N, Hochhauser D, Shiu KK et al. (2020) Outcomes of the 2019 novel coronavirus in patients with or without a history of cancer – a multi-centre North London experience. medRxiv 2020.2004.2016.20061127.

10. Dai M, Liu D, Liu M et al. (2020) Patients with cancer appear more vulnerable to SARS-CoV-2: a multicenter study during the COVID-19 outbreak. Cancer Discov 10(6):783–791. doi: 10.1158/2159-8290.Cd-20-0422.

11. Miyashita H, Mikami T, Chopra N et al. (2020) Do patients with cancer have a poorer prognosis of COVID-19? An experience in New York City*.* Ann Oncol 31:1088–1089. doi: 10.1016/j.annonc.2020.04.006.

12. Aboueshia M, Hussein MH, Attia AS et al. (2021) Cancer and COVID-19: analysis of patient outcomes. Future oncol 17(26), 3499–3510. https://doi.org/10.2217/fon-2021-0121

13. Saini KS, Tagliamento M, Lambertini M, et al. (2020) Mortality in patients with cancer and coronavirus disease 2019: a systematic review and pooled analysis of 52 studies. Eur J Cancer 139:43-50. doi:10.1016/j.ejca.2020.08.011.

14. Colorado Department of Public Health & Environment. Welcome to the Colorado COVID Symptom Support tool. Accessed 5 Sep 2020. https://intake-app-dot-cdphe-erm.appspot.com/intake-form

15. Hargittai E, Redmiles E. Will Americans be willing to install COVID-19 tracking apps? *Scientific American Observations* blog. April 28, 2020. Accessed 5 Sep 2020. https://blogs.scientificamerican.com/observations/will-americans-be-willing-to-install-covid-19-tracking-apps/

16. Drew DA, Nguyen LH, Steves CJ, et al. (2020) Rapid implementation of mobile technology for real-time epidemiology of COVID-19. Science 368(6497):1362-1367. doi:10.1126/science.abc0473.

17. Holshue ML, DeBolt C, Lindquist S, et al. (2020) First case of 2019 novel coronavirus in the United States. N Engl J Med 382(10):929-936. doi:10.1056/NEJMoa2001191.

18. Mooney K, Berry DL, Whisenant M, et al. (2017) Improving cancer care through the patient experience: how to use patient-reported outcomes in clinical practice. Am Soc Clin Oncol Educ Book 37:695-704. doi:10.14694/EDBK_175418.

19. Aaronson N, Elliott T, Greenhalgh J, et al. User's guide to implementing patient-reported outcomes assessment in clinical practice: version 2. January 2015. Accessed 5 Sep 2020. https://www.isoqol.org/wp-content/uploads/2019/09/2015UsersGuide-Version2.pdf

20. Jacks T, Jaffee E, Singer D, Cancer Moonshot Blue Ribbon Panel. Cancer Moonshot Blue Ribbon Panel Report 2016. October 17, 2016. Accessed 5 Sep 2020. https://www.cancer.gov/research/key-initiatives/moonshot-cancer-initiative/blue-ribbon-panel/blue-ribbon-panel-report-2016.pdf

21. Cleeland CS, Mendoza TR, Wang XS, et al. (2000) Assessing symptom distress in cancer patients: the M.D. Anderson Symptom Inventory. Cancer 89(7):1634-1646. doi:10.1002/1097-0142(20001001)89:7<1634::aid-cncr29>3.0.co;2-v.

22. Herdman M, Gudex C, Lloyd A, et al. (2011) Development and preliminary testing of the new five-level version of EQ-5D (EQ-5D-5L). Qual Life Res 20(10):1727-1736. doi:10.1007/s11136-011-9903-x.

23. Pickard AS, Law EH, Jiang R, et al. United States Valuation of EQ-5D-5L Health States Using an International Protocol. Value Health. 2019 Aug;22(8):931-941. doi: 10.1016/j.jval.2019.02.009.

24. Sloan JA, Vargas-Changes D, Kamath CC, et al. (2003) Detecting worms, ducks, and elephants: a simple approach for defining clinically relevant effects in quality-of-life measures. J Cancer Integr Med 1:41-47.

25. Cohen J. (1988) Statistical Power Analysis for the Behavioral Sciences. Hillsdale, New Jersey: Lawrence Erlbaum Associates.

26. Centers for Disease Control and Prevention. Coronavirus Disease 2019 (COVID-19) - Symptoms. Updated May 13, 2020. Accessed 5 Sep 2020. https://www.cdc.gov/coronavirus/2019-ncov/symptoms-testing/symptoms.html

27. Galván Casas C, Català A, Carretero Hernández G, et al. (2020) Classification of the cutaneous manifestations of COVID-19: a rapid prospective nationwide consensus study in Spain with 375 cases. Br J Dermatol 183(1):71-77. doi:10.1111/bjd.19163.

28. Zhang X, Chen X, Chen L, et al. (2020) The evidence of SARS-CoV-2 infection on ocular surface. Ocul Surf 18(3):360-362. doi:10.1016/j.jtos.2020.03.010.

29. Streiner DL (2003) Starting at the beginning: an introduction to coefficient alpha and internal consistency. J Pers Assess 80(1):99-103. doi:10.1207/S15327752JPA8001_18.

30. Matheson GJ (2019) We need to talk about reliability: making better use of test-retest studies for study design and interpretation. PeerJ 7:e6918. doi:10.7717/peerj.6918.

31. Wilson IB, Cleary PD (1995) Linking clinical variables with health-related quality of life. A conceptual model of patient outcomes. JAMA 273(1):59-65.

32. Mendoza TR, Wang XS, Lu C, et al. (2011) Measuring the symptom burden of lung cancer: the validity and utility of the lung cancer module of the M. D. Anderson Symptom Inventory. Oncologist 16(2):217-227. doi:10.1634/theoncologist.2010-0193.

33. Kirkova J, Davis MP, Walsh D, et al. (2006) Cancer symptom assessment instruments: a systematic review. J Clin Oncol 24(9):1459-1473. doi:10.1200/JCO.2005.02.8332.
